# Chitosan Effect on Hardening Dynamics of Calcium Phosphate Cement: Low-Field NMR Relaxometry Investigations

**DOI:** 10.3390/polym14153042

**Published:** 2022-07-27

**Authors:** Ioana Lacan, Mărioara Moldovan, Codruța Sarosi, Ioan Ardelean

**Affiliations:** 1 Department of Physics and Chemistry, Technical University of Cluj-Napoca, 400114 Cluj-Napoca, Romania; ioana_lacan@yahoo.com; 2 Department of Polymer Composites, “Raluca Ripan” Chemistry Research Institute, “Babes-Bolyai” University, 400294 Cluj-Napoca, Romania; marioara.moldovan@ubbcluj.ro (M.M.); codruta.sarosi@gmail.com (C.S.)

**Keywords:** bone cements, calcium phosphate, chitosan, hardening, NMR relaxometry

## Abstract

Calcium phosphate cements are used in dentistry and orthopedics to repair and reconstruct bone defects. The properties of these bone cements can be improved by introducing additives into their composition. One favorable additive is chitosan, which can be beneficial but can also cause considerable damage if it has a high load, thus, limiting its clinical applicability and performance. That is why understanding chitosan’s role in cement composition is an important issue when developing new materials. The present work uses low-field nuclear magnetic resonance (NMR) relaxometry to investigate the effect introduced by the addition of chitosan on the hardening process of calcium phosphate cement. Two samples, prepared with and without chitosan, were comparatively investigated during the first six minutes of hardening. The liquid evolution inside these samples was monitored using transverse relaxation time distributions. It demonstrated an acceleration effect on the hardening dynamics introduced by the presence of chitosan. Furthermore, it was shown that even after one hour of hardening, there were still unreacted monomers inside the bone cement and their amount was reduced in the presence of chitosan.

## 1. Introduction

Bone cements are bio-materials applied for the reconstruction of bone defects and bone tissue regeneration [1]. According to their chemical composition, bone cements can be classified into acrylic bone cements (ABCs) and calcium phosphate cements (CPCs) [2]. Acrylic bone cements are bio-compatible and help to quickly fix the implant to the bone, having an essential role in orthopedic surgery for fixing artificial joints. However, acrylic bone cements also have some disadvantages, such as a lack of bio-activity, high exothermic temperature and monomeric toxicity [3,4]. To overcome these limitations, calcium phosphates have been used in the last few years as bio-materials in the composition of state-of-the-art bone cements.

Calcium phosphate cements have a chemical composition similar to that of natural bone, which facilitates osteoconductivity and osteogenesis [5]. The most widely used calcium phosphate is tricalcium phosphate (TCP), which is a commercially available material obtained by treating hydroxyapatite with phosphoric acid and slaked lime using double decomposition reactions under controlled pH conditions [6,7]. It can be found in three polymorphic crystalline phases, denoted alpha (α-TCP and α’-TCP) [6], which is stable at elevated temperatures and has a better solubility in aqueous solutions, and beta (β-TCP) respectively, which is one of the most-used synthetic bone graft substitutes [7].

TCP-based bio-materials are routinely used in various medical applications [2,5], but these synthetic materials also have some drawbacks, such as poor mechanical behavior, and a bio-compatibility that is more limited than that of natural polymers, such as chitosan (CS) [8,9,10]. That is why there are many reports in the literature on the introduction of chitosan in CPCs, resulting in the improvement of certain properties, such as osseointegration and bio-compatibility [10,11,12,13,14]. Note, however, that the incorporation of chitosan as an additive may be beneficial [14] compared to other organic materials, but it can also cause considerable damage to the mechanical properties of cements when overloaded, thus, limiting certain clinical uses. That is why the understanding of chitosan’s role in bone cement composites is important and worth being investigated.

A technique with a high potential for investigating bone cements is low-field NMR relaxometry [15,16]. This technique was successfully applied to characterize the evolution of complex porous structures, as, for instance, composites based on Portland cement [16,17]. NMR relaxometry was successfully used to monitor the influence of different additives and admixtures on hydration dynamics and pore development in such materials. With respect to medical applications, NMR relaxometry was successfully used for detecting pre- and post-damage microstructural changes in human cortical bones [18] and for the detection of age-induced structural changes in human teeth [15]. Low-field NMR relaxometry investigations have the advantages of being completely non-invasive and can be applied without any special preparation of the sample. Consequently, the evolution of the sample can be monitored during its transformation from a gel to a solid. In the present work, NMR relaxometry was used again to extract information on the effects introduced by incorporating chitosan into tricalcium phosphate bone cements. Thus, the influence of chitosan on the hardening dynamics and the evolution of the sample from a gel-like material to a solid matrix were investigated.

## 2. Materials and Methods

### 2.1. Sample Preparation

Two calcium phosphate cements, denoted C1 and C2, respectively, were prepared by mixing two sterile components of an organic phase and inorganic phase, as indicated in Table 1. In one component, an initiator, benzoyl peroxide (BPO), was added, and in the other component, an accelerator, N N dihydroxyethyl-p-toluidine (DHEPT), was added. When the two components were mixed, the liquid monomers polymerized around the powder particles to form hardened cements at room temperature. The two bone cements contained the same amounts of liquid and chemical initiator, and the only difference was given by the partial replacement of TCP with chitosan in the C2 cement. The chitosan used in the preparation of the cement composite material had a high degree of deacetylation (>85%) and was manufactured by Sigma-Aldrich (Sigma-Aldrich Inc., St. Louis, MO, USA).

The liquid phase of the cement was composed of urethane dimethacrylate (UDMA)—an organic monomer with a large and rigid structure, which decreases the polymerization shrinkage; hydroxyethyl methacrylate (HEMA)—a viscosity modifier; and a synthetic polyethylene glycol polymer (PEG 400) with a low molecular weight; therefore, it is used as a solvent for a large number of substances that easily dissolve into water, but also as a solubilizing agent for active substances and excipients in liquid and semi-solid preparations, due to its low toxicity [19]. The incorporation of PEG improved the workability of the brushite cement without producing inadequate modifications in the physicochemical and biological properties [20].

In the case of the C1 cement, the inorganic component was composed of bio-active, bio-compatible and bio-degradable tricalcium phosphate (TCP), while in the case of the C2 cement, the TCP filler was partially replaced with chitosan (CS) nanoparticles acting as anti-microbial agents to reduce the bacterial infection of bone cements. The initial (<1 min) polymerization reaction took place at an ambient temperature of 23 °C and a humidity of 53%. The reaction time of the polymerization, in which the sample evolved from the liquid phase to a gel state and then to a hardened material, was between 3 and 3.5 min, and the reaction was exothermic.

### 2.2. NMR Relaxometry Technique

Transverse NMR relaxation measurements were performed using a low-field instrument (Minispec MQ20, Bruker, Karlsruhe, Germany) operating at a 20 MHz proton resonance frequency. The measuring temperature was set at 35 °C; close to body temperature. The well-known Carr–Purcell–Meiboom–Gill (CPMG) [21] pulse sequence was implemented for performing the measurements. The CPMG pulse sequence consisted of a series of 180-degree radiofrequency (RF) pulses following a single 90-degree RF pulse. This sequence generated a train of spin echoes, separated by an echo time interval, 2τ. The amplitudes of these echoes were attenuated by transverse relaxation phenomena. In the case of a heterogeneous sample, consisting of separate spin reservoirs, which are characterized by distinct transverse relaxation times, *T*_2_, the *n*-th echo in the echo train attenuated according to the following formula:

(1) $$A_{n}=A_{0}\int_{0}^{\infty }P\left(T_{2}\right)e^{-\frac{2n\tau }{T_{2}}}dT_{2},$$

where, $A_{0}$ is a constant depending on the size of the sample and the technical characteristics of the NMR instrument. $P\left(T_{2}\right)$ is a relaxation time probability density. The above formula suggests that a numerical Laplace inversion of the recorded CPMG echo train allows for the extraction of the relaxation time probability density [22,23].

In the case of molecules confined inside porous materials, as, for instance, bone cements, the relaxation time *T*_2_ could be related to the pore size with the following formula:

(2) $$\frac{1}{T_{2}}=\rho \frac{S}{V}=\rho \frac{3}{R},$$

where, ρ is the pore surface relaxivity constant, S/V is the surface-to-volume ratio and R is the pore radius, assuming spherical pores. The above equation shows that a direct relation can be established between the pore size distribution and the relaxation time distribution, provided that no exchange exists between different pore reservoirs during the echo time interval. Note, however, that the above Equation (2) disregards the diffusion effects on the echo attenuation [16].

In our hardening investigations on bone cement, the CPMG echo trains were consecutively recorded every 30 s up to 6 min. The first echo train was acquired at 1 min from the mixing start, immediately after the introduction of the sample paste inside the NMR tube. The echo time interval of 0.1 ms was implemented in all of the measurements to reduce diffusion effects on the echo train attenuation [16]. The repetition time was also kept at a minimum of 0.5 s to reduce the total duration of the CPMG experiment to less than 5 s. This assured no sample changes during the experiment.

## 3. Results and Discussion

**Figure 1:**
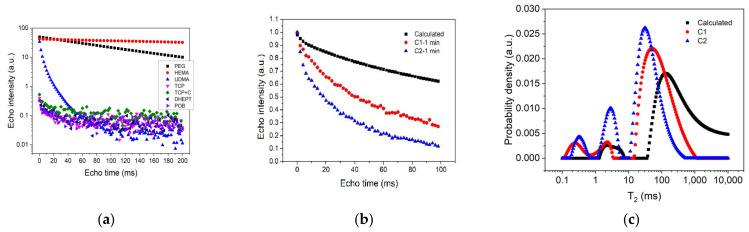
(**a**) CPMG echo trains recorded for the ingredients constituting the two bone cements, as indicated in the legend; (**b**) CPMG echo trains recorded for the two cement pastes C1 (circles) and C2 (triangles) at one minute from the mixing start point. The calculated curve, obtained as a weighted summation of the liquid components, is also indicated (squares). (**c**) Relaxation time distributions extracted from the CPMG echo trains shown in Figure 1b.

To facilitate the interpretation of the NMR data, the relaxation measurements on the mixing ingredients were performed first. Figure 1a shows the CPMG echo series recorded on the bulk substances used in the composition of cement samples C1 and C2, respectively (see Table 1). Note that all the data were normalized by the mass of the measured sample. The first three substances (PEG, HEMA and UDMA) had mobile molecules, were rich in protons, and, therefore, revealed high NMR signals and long relaxation times. The rest of the components were solid-like and were characterized by short transverse relaxation times (in the range of milliseconds). Furthermore, a weaker NMR signal was detected, which reached the noise level after 40 ms. The long relaxation times corresponding to HEMA and PEG indicated that the molecules of these components were mobile, as is specific for bulk liquids. In the case of UDMA, a non-exponential echo train decay was observed, which indicated a more complicated internal structure, specific to viscous polymers. Considering the above observations, we concluded that, in the case of the C1 and C2 cement composites, the recorded NMR signal in the CPMG pulse sequence (Figure 1b) mainly originated in the liquid-like ingredients. That is why only these ingredients were considered in our analysis.

For a further demonstration of the above conclusion, we calculated the weighted summation of the relaxation decay curves corresponding to PEG, HEMA and UDMA, considering the proportions indicated in Table 1. The result of these calculations is indicated in Figure 1b (squares). One can observe a similar shape with those recorded at 1 min from the beginning of the mixing process on cement mixtures C1 and C2, respectively. However, the higher slope detected in the case of the C1 and C2 cements, as compared with the calculated slope, indicated that the polymerization process already started during the mixing time (<1 min). One can also observe a faster evolution of the cement sample containing chitosan (C2) as compared to the one without chitosan (C1).

The relaxation time distributions (Figure 1c) extracted from the echo decays (Figure 1b) confirmed the above observations. They showed the presence of two peaks in the case of the calculated curve (squares) and three peaks in the case of the cement samples (circles and triangles). For the calculated curve, the smaller peak (first from the left) corresponded to UDMA, while the larger one could be associated with an overlapped contribution from PEG and HEMA. The same two peaks could also be observed in the case of the two cement composites, but with the larger peak shifted to smaller relaxation times and an extra peak arising at approximately 0.2–0.8 ms (first from the left). The latter could be attributed to a compound with a viscous structure and low mobility. These distributions evolved during the hardening process, toward overlapping.

**Figure 2:**
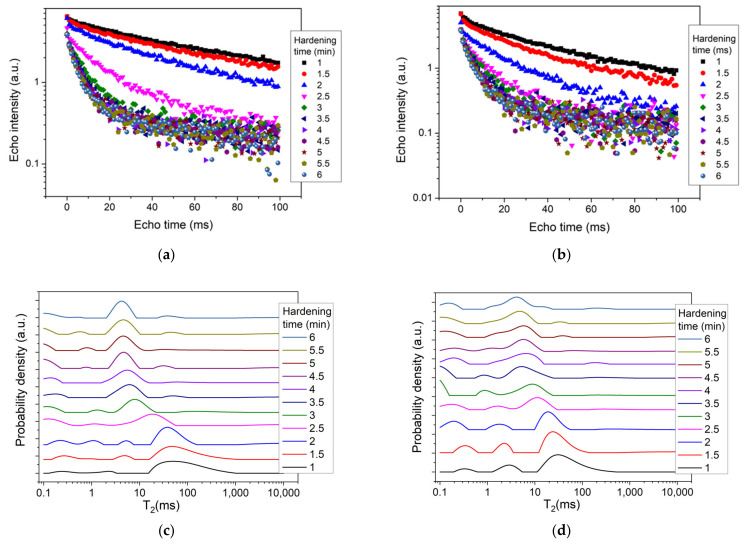
(**a**,**b**) CPMG echo trains recorded during the first 6 min of the polymerization process for the two bone cements, C1 (**a**) and C2 (**b**), respectively. (**c**,**d**) Relaxation time distributions during the hardening of the two bone cements C1 (**c**) and C2 (**d**), respectively. The relaxation distributions were extracted from the CPMG echo trains (**a**,**b**). The hardening times are indicated in the legend.

To monitor the acceleration effects introduced by the presence of chitosan on cement hardening, multiple CPMG echo trains were recorded on each sample at 30 min intervals. The results are depicted in Figure 2a, b, respectively. One can observe an increase in the slope of these curves during the first 3.5 min of polymerization, representing the initial setting time of the bone cement [24]. It was also directly observed that the two samples revealed different hardening dynamics. For a better monitoring of the chitosan effects on polymerization dynamics, we extracted from the CPMG echo trains depicted in Figure 2a,b the corresponding relaxation time distributions via a numerical Laplace inversion [22]. The corresponding relaxation time distributions, at different hardening times, are shown in Figure 2c,d.

By analyzing the relaxation time distributions at 1 min from the mixing start point (the shortest accessible in our measurements), one can observe a series of three peaks (Figure 2c,d). The first peak from the left, arising during the 0.1–1 ms interval, could be attributed to the already formed polymerization products in the cement microstructure, which occurred in the form of a solid network [25]. As can be seen, this peak shifted to the left during the polymerization process, withdrawing from the detection interval and indicating the development of a solid component. The second peak from the left, arising between 1 ms and 10 ms, may be attributed to the UDMA component and its area reducing during hardening. The larger peak, arising between 10 ms and 1000 ms (depending on the cement sample), may be attributed to the liquid components (PEG and HEMA). Its evolution during hardening revealed a consumption of the mobile component and a shift to smaller values, as polymerization progressed. This evolution could be associated with the transformation of components during cement setting due to gelation reactions [26].

**Figure 3:**
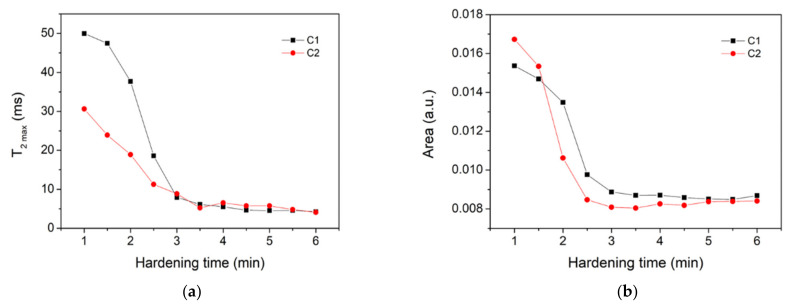
(**a**) Evolution of the relaxation time corresponding to the peak maximum in the case of liquid-like component (large peak in Figure 2) versus hardening time. (**b**) Area under the curve for the distributions shown in Figure 2, calculated for relaxation times *T*_2_ > 1 ms. The two samples under investigation are indicated in the legend.

Figure 3a depicts the position of the peak maximum corresponding to the most mobile component versus hardening time. One can observe a decay of the relaxation time for both samples (C1 and C2) with a plateau starting at 3.5 min, which could be considered as the final setting time of the cement material [24]. The presence of a plateau showed that the polymerization process almost stopped, and that there was no change inside the two samples. When we analyzed the *T*_2_ values, we noticed smaller relaxation times for the sample containing chitosan (C2) as compared to the sample without chitosan (C1). According to Equation (2), this behavior could be attributed to smaller pores forming inside the C2 sample as compared to the C1 sample.

Figure 3b shows the area under the curves in Figure 2c,d, respectively, calculated in the region with relaxation times *T*_2_ > 1 ms. The evolution of the area indicated a faster consumption of UDMA, PEG and HEMA components for the cement containing chitosan (C2) as compared to that without chitosan (C1). Note, however, that a clear separation between UDMA’s contribution and PEG and HEMA’s contributions to the area was not feasible. The relaxation time distributions also showed a continuous transformation of the liquid components into less mobile components (shorter relaxation times).

**Figure 4:**
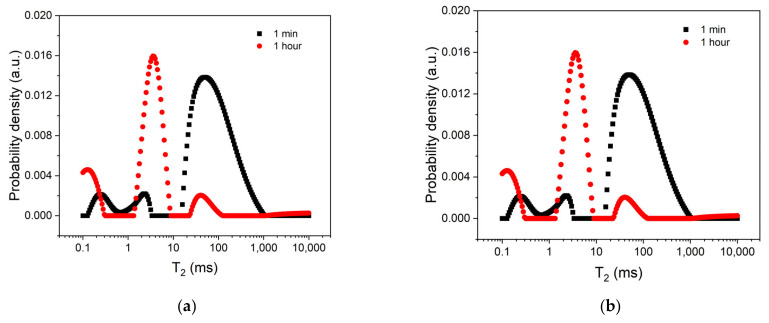
The relaxation time distributions for the two bone cements C1 (**a**) and C2 (**b**), respectively. Two hardening times were considered, as indicated in the legend.

Figure 4 compares the relaxation time distributions of the two samples after 1 h of hardening with those recorded at 1 min. For the distribution recorded at 1 h (circles), one can clearly distinguish the presence of three peaks, associated with the three spin reservoirs. The first peak, arising between 0.1 ms and 0.3 ms, could be associated with a solid-like component, but with a certain degree of mobility. The second peak, arising between 1 ms and 10 ms, could be associated with a viscous component characterized by a higher degree of mobility than the solid-like component (peak at 0.1–0.3 ms). This peak originated in the liquid component (larger peak) observed at 1 min of hardening (squares). The third peak, arising between 10 ms and 100 ms, may be attributed to the unreacted monomers. If we compared the area of the larger peak at 1 min with the area of the third peak at 1 h, we could estimate the proportion of unreacted monomers. Thus, this comparison gave us 5.8% of an unreacted monomer in the case of the C1 sample and 4.3% in the case of the C2 sample. Consequently, it demonstrated the beneficial effect of chitosan in reducing the amount of unreacted monomers.

## 4. Conclusions

The hardening dynamics of the two bone cements, manufactured with and without chitosan, were investigated in a non-invasive manner via low-field NMR relaxometry. The continuous transformation of the materials from a viscous structure to a solid matrix was monitored, and the hardening time was determined from relaxation time distributions. Using the CPMG technique, it was possible to study the transformation of the sample starting at 1 min after mixing the ingredients, and up to 6 min. It was observed that the final hardening time of the cement sample was not influenced by the presence of chitosan, but the hardening dynamics were accelerated by the partial replacement of TCP with chitosan. Moreover, the evolution of the liquid UDMA and PEG into components with lower mobility was monitored, and the presence of residual monomers could be quantified. It can be concluded that the presence of chitosan slightly reduced the quantity of unreacted monomers. The results obtained here may be useful for understanding the hardening dynamics and provide a toll in the design of new calcium phosphate cements.

## Figures and Tables

**Table 1:** Composition of the two bone cements: C1 and C2.

Cement	Organic Phase (%)			Inorganic Phase (%)		Initiation System (%)	
	UDMA	HEMA	PEG 400	TCP	Chitosan	BPO	DHEPT
C1	3	25	22	50	0	2	0.75
C2	3	25	22	25	25	2	0.75

UDMA: 1,6-bis(methacryloxy-2-ethoxycarbonylamino)-2,4,4-trimethylhexane (Sigma Aldrich, St. Louis, MO, USA); HEMA: 2-hydroxyethyl methacrylate (Alfa Aesar, Haverhill, MA, USA)*;* PEG: polyethylene glycol (Sigma Aldrich, St. Louis, MO, USA); TCP: tricalcium phosphate (Merck, Darmstadt, Germany); POB: benzoyl peroxide (Merck, Darmstadt, Germany); DHEPT: dihydroxyethyl-p-toluidine (Sigma Aldrich, St. Louis, MO, USA).

## Data Availability

Data may be provided on request from corresponding author.
